# Cardiovascular reactions to acute psychological stress and academic achievement

**DOI:** 10.1111/psyp.14064

**Published:** 2022-03-30

**Authors:** Annie T. Ginty, Alexandra T. Tyra, Danielle A. Young, Ryan C. Brindle, Susanne R. de Rooij, Sarah E. Williams

**Affiliations:** ^1^ Department of Psychology and Neuroscience Baylor University Waco Texas USA; ^2^ Department of Cognitive and Behavioral Science & Neuroscience Program Washington and Lee University Lexington Virginia USA; ^3^ Department of Epidemiology and Data Science, Amsterdam University Medical Centers University of Amsterdam Amsterdam Netherlands; ^4^ School of Sport, Exercise, and Rehabilitation Sciences University of Birmingham Birmingham UK

**Keywords:** acute psychological stress, cardiovascular reactivity, cognitive function

## Abstract

Cardiovascular reactions to acute psychological stress have been associated with cognitive function. However, previous work has assessed cardiovascular reactions and cognitive function in the laboratory at the same time. The present study examined the association between cardiovascular reactions to acute psychological stress in the laboratory and academic performance in final year high school students. Heart rate, blood pressure, stroke volume, and cardiac output reactions to an acute psychological stress task were measured in 131 participants during their final year of high school. Performance on high school A‐levels were obtained the following year. Higher heart rate and cardiac output reactivity were associated with better A‐level performance. These associations were still statistically significant after adjusting for a wide range of potentially confounding variables. The present results are consistent with a body of literature suggesting that higher heart rate reactions to acute psychological stress are associated with better cognitive performance across a variety of domains.

## Abstract 2

The present study is the first to examine the associations between cardiovascular reactions to stress in the laboratory and academic achievement. Additionally, it is the first to examine a more comprehensive hemodynamic profile of cardiovascular reactivity (e.g., cardiac output) with cognitive function. The present results are consistent with a body of literature suggesting that higher heart rate reactions to acute psychological stress are associated with better cognitive performance across a variety of domains.

## 1 INTRODUCTION

The cardiovascular stress reactivity hypothesis postulates that exaggerated cardiovascular reactions to acute psychological stress are associated with elevated risk for pre‐clinical and clinical endpoints of cardiovascular disease (for reviews see, Chida & Steptoe, 2010; Gerin et al., 2000; Krantz & Manuck, 1984; Lovallo, 2005; Taylor et al., 2003; Treiber et al., 2003; Turner et al., 2020). Indeed, there is robust cross‐sectional and prospective research demonstrating an association between exaggerated cardiovascular stress reactivity and the development of high blood pressure (BP) and hypertension (e.g., Carroll et al., 2011, 2012; Chida & Steptoe, 2010). Higher BP is associated with reduced cognitive performance among children and younger adults (Waldstein et al., 1996; Walker et al., 2017) and cognitive decline in adult samples (Gasecki et al., 2013; Gottesman et al., 2014; Hughes & Sink, 2016; Iadecola et al., 2016; Kohler et al., 2014). Similarly, hypertension is associated with poorer cognitive function (Elias et al., 2012; Friedman et al., 2014; Gorelick et al., 2011; Iadecola et al., 2016; King & Miller, 1990; Launer et al., 1995; Singh‐Manoux et al., 2005; Tzourio et al., 2014; Waldstein, 2003). If excessive cardiovascular reactivity contributes to hypertension and hypertension is associated with poorer cognitive function, then it would be expected that reactivity would be negatively associated with cognitive ability (i.e., higher cardiovascular reactivity would be related to poorer cognitive function).

However, early research examining the association between cardiovascular stress reactivity and cognitive ability has produced equivocal results. A study in infants demonstrated that a higher suppression of a heart period‐based index of vagal tone was related to greater maturity of cognitive skills and greater coordination of motor behaviors (DeGangi et al., 1991). In a young adult sample, sympathetic activity was negatively associated with task performance, whereas an index of vagal tone was positively related to performance (Duschek et al., 2009). Two other studies in young (Backs & Seljos, 1994) and older (Wright et al., 2005) adults found no association between cognitive function and cardiovascular stress reactivity to cognitive tasks. The lack of consensus from these early studies may be due to differences in populations studied, physiological parameters measured, and cognitive tasks employed. Perhaps more importantly, all of these studies measured cognitive ability as performance on the stress reactivity challenge. Using measures of cognitive ability that are independent of the mental stress task employed to elicit reactivity would provide a stronger, and less confounded, test of the association between cognitive ability and cardiovascular stress reactivity.

More recent research has extended previous work examining the relationship between cognitive ability and cardiovascular stress reactivity by using acute psychological stress tasks independent of the cognitive ability assessments (i.e., the acute psychological stress task is not also the cognitive test in which cognitive function is being measured from). The majority of cross‐sectional evidence from research in samples across the lifespan conclude that higher cardiovascular and/or cortisol stress reactivity is associated with better cognitive function (Gao, Borlam, & Zhang, 2015; Ginty, Phillips, Roseboom, et al., 2012; Lin et al., 2014; Slattery et al., 2013; Wawrzyniak et al., 2016). However, there have been two studies demonstrating that higher reactivity to stress is associated with worse cognitive performance (Brown et al., 2009; Hendrawan et al., 2012). Prospective research suggests that there may be a bi‐directional relationship between cognitive ability and cardiovascular reactivity. In a sample of 409 middle aged adults, general intelligence scores and simple reaction time predicted cardiovascular stress reactivity 7‐years later (Ginty, Phillips, Der, Deary, & Caroll, 2011). Two separate studies support a relationship in the opposite direction (i.e., cardiovascular stress reactivity predicting cognitive function; Ginty, Phillips, Der, Deary, & Carroll, 2011; Yano et al., 2016). In a study of 3021 participants, lower cardiovascular reactivity to acute psychological stress measured in young adulthood predicted worse cognitive function, indexed by the Digit Symbol Substitution Test and the Stroop, 23‐years later (Yano et al., 2016). Similarly, a study of 1647 adults demonstrated that lower cardiovascular reactivity to acute psychological stress was associated with greater cognitive decline, as measured by changes in choice reaction times, over a 7‐year period (Ginty, Phillips, Der, Deary, & Carroll, 2011). Overall, a growing body of evidence suggests that higher cardiovascular stress reactivity may be associated with better cognitive function. Interestingly, these results suggest this relationship is not just apparent for general intelligence, but spans multiple domains of cognitive function (i.e., working memory, attention, executive function, and fluid intelligence). However, previous studies have primarily focused on blood pressure and heart rate responses to stress and have not included more comprehensive hemodynamic measures such as stroke volume or cardiac output. If the relationship between cardiovascular stress reactivity and cognitive function is robust, then there should be a significant association between cognition with these more comprehensive measures (e.g., cardiac output).

To our knowledge, no study to date has examined the relationship between cardiovascular reactivity to an acute laboratory stress task and performance on a “real‐life” assessment. Measuring the association between cardiovascular stress reactivity and cognitive performance independently from acute psychological stress tasks has advanced knowledge of the relationship between stress reactivity and cognitive function. If the relationship between cardiovascular reactivity and cognitive ability is truly robust, one would expect cardiovascular reactivity in the laboratory to predict performance on a “real‐life” cognitive assessment.

General Certificate of Education Advanced Level Qualifications (GCE A‐Levels) are subject‐based high school qualifications used for standard assessment of prospective pre‐university students in England, Wales, and Northern Ireland. Longitudinal work demonstrates that GCE A‐level performance is a stronger predictor of academic and career success compared to general intelligence tests (McManus et al., 2003). To achieve high scores on GCE A‐Levels, students need to have high levels of general academic knowledge (O’Hare & McGuinness, 2009), possess strong critical thinking skills (O’Hare & McGuinness, 2015), and display high levels of motivation (McManus et al., 2005). Strong performance in multiple cognitive domains are required to achieve high grades and many of these domains (general intelligence, working memory, attention, fluid intelligence) have been associated with cardiovascular reactivity (Gao et al., 2016; Ginty, Phillips, Der, Deary, & Caroll, 2011; Ginty, Phillips, Der, Deary, & Carroll, 2011; Ginty, Phillips, Roseboom, et al., 2012; Lin et al., 2014; Yano et al., 2016). Interestingly, in addition to poorer cognitive function, lower cardiovascular stress reactivity has also been associated with reduced levels of motivation and behavioral engagement (Chauntry et al., 2019; Ginty et al., 2015; Ginty et al., 2020; Messay & Marsland, 2015; Whittaker & Chauntry, 2021).

The present study aimed to examine the relationship between cardiovascular stress reactivity in a sample of final‐year high school students and performance on the GCE A‐Levels. Based on the balance of prior research, it was hypothesized that greater cardiovascular stress reactivity to acute psychological stress would be associated with better GCE A‐Level performance.

## 2 METHOD

### 2.1 Participants

Final year high school students (*N* = 185) were recruited from 28 high schools in Birmingham, England to participant in an in‐person laboratory visit (Phase 1) between October 2012 and April 2013. Participants were recruited through visits by the investigators attending assemblies at their high school and announcing the study. Heart rate and blood pressure data from nine participants were unusable due to signal acquisition errors. Students completed their A‐level examinations in June 2013. GCE A‐Level scores were obtained via questionnaire at phase 2 follow‐up (April 2014). Forty‐four participants failed to complete the second phase (Phase 2) and did not provide A‐Level scores (Ginty et al., 2015) and one participant who completed Phase 2 did not provide A‐Level scores. The final sample included 131 participants with full heart rate (HR) and blood pressure (BP) data. See Table 1 for participant demographic information. Power analyses using G*Power (80% power, α = 0.05) to test the *R* ^2^ value and to test for the individual regression slopes with 11 predictors suggested a minimum sample of 123 was needed to detect a moderate effect size. Exclusion criteria included a history of cardiovascular disease. Participants were asked to refrain from vigorous exercise and alcohol 12 h, caffeine 2 h, and food and drink other than water 1 h prior to testing. All participants and legal guardians, if participants were under 18, provided informed consent. Participants received £10 for study participation. The study was approved by the University Ethics Committee.

**TABLE 1:** Demographic information of participants at phase 1

Variable	Mean/number	*SD*/%	Range
Gender (% female)	112	84.20	
Ethnicity (% White)	74	55.60	
Age	17.99	0.42	17.02–19.47
SES	2.62	1.70	1.00–7.00
HADS‐depression score	4.50	3.26	0.00–14.00
Total A‐Level score	340.30	114.55	40.00–700.00

### 2.2 Acute psychological stress task

The acute psychological stress task was completed during Phase 1. Participants completed the Paced Auditory Serial Addition Task (PASAT; Gronwall, 1977), which has been shown to significantly perturb cardiovascular activity (Mathias et al., 2004; Ring et al., 2002) and demonstrates both high levels of internal consistency and good test–retest reliability (Ginty et al., 2013; Willemsen et al., 1998). Participants listened to a recording with a series of single‐digit numbers. Participants were instructed to add consecutive numbers together, verbalize their response, and simultaneously remember the most recently stated number in order to add it to the following stated number. The interval between numbers was 2.4 s for the first minute of the task and decreased .4 s each minute until completion of the task. The task included additional elements to increase feelings of stress (Ginty, Phillips, Higgs, et al., 2012). Participants were told they were in direct competition with their peers and a scoreboard was visible in the laboratory. Additionally, participants were told they were being videotaped and told the recording would be assessed by “body language experts.” In reality, no such recordings or assessments were made. During the stress task, a research assistant stood at approximately 0.25 m and the participant was told they would hear a loud, aversive beep every time they gave an incorrect answer, stuttered, mumbled, or hesitated. However, beeps were standardized for all participants and given approximately every 10 numbers. Lastly, participants were instructed to look at themselves in a mirror placed 0.5 m away for the duration of the task.

### 2.3 Cardiovascular measurements and data processing

Standard electrocardiography (ECG) was used to continuously measure HR using a 3‐lead configuration with electrodes placed on the lower left rib and the right and left clavicle. Raw ECG data were collected using a Grass P511 amplifier (Grass Instruments, USA), CED Power 1401 analog to digital converter, and Spike 2 software at a sampling frequency of 1000 Hz. Each trace was visually inspected for artifacts and Kubios HRV (Tarvainen et al., 2014) was used to calculate average HR for each 10‐min formal baseline and stress period (calculated from inter‐beat intervals). Systolic blood pressure (SBP) and diastolic blood pressure (DBP) were measured discontinuously (2, 4, 6, 8‐min of each period) via a semi‐automatic sphygmomanometer (Critkon Inc., Tampa, FL). Echocardiographic measurements were performed using a Philips Sonos 7500 ultrasound machine with an S3 two‐dimensional transducer (1–2 MHz). Digital images of spectral waveforms were recorded discontinuously for later analysis. An apical five‐chamber view of the heart was used with Doppler mode to identify flow through aortic valve during systole. The velocity profile of aortic flow was obtained using pulse wave spectral mode at a screen sweep speed of 100 mm s^−1^, with Doppler sampling of the flow taken immediately below the orifice of the aortic valve. The flow was quantified automatically using the velocity time integral, which is the mean distance through which blood travels in the outflow tract during ventricular contraction. Each velocity time integral was made from at least three velocity profiles taken toward the end of expiration. For each measurement point, averages were obtained from three or more spectral waveforms and measurements for aortic blood flow were averaged across 60‐s intervals. The diameter of the aortic valve was measured from a parasternal long axis view and the aortic valve area was calculated. Stroke volume (SV) was calculated by multiplying the velocity time integral by the aortic valve area. Cardiac output (CO) was calculated as HR x SV. CO data were unavailable for 24 participants due to image acquisition difficulties, resulting in a sample size of 108 for all analyses of CO and SV. There were no demographic, heart rate, or blood pressure differences between those who had full CO and SV data and those who did not. A study comparing measurements of CO using impedance cardiography and Doppler echocardiography concluded that the latter provided a more reliable and clinically acceptable and accurate method of measuring cardiac during hemodynamic challenge (Fellahi et al., 2009).

### 2.4 Total A‐Level score

In June 2013, students completed their General Certificate of Education (GCE A‐Level) examinations. GCE A‐Levels are school‐leaving qualifications in the United Kingdom and are recognized as the standard for assessing appropriateness for admission to university. In 2013, overall A‐level scores were obtained through a mixture of coursework and examinations. They are standardized across the country. Students are often provided conditional offers to universities, which are dependent on achieving a minimum set of final grades. Students choose subjects they will study for the remaining two years of school (i.e., sixth form) and the GCE A‐Level examinations are over their chosen subjects. A variety of subjects are offered, such as: English Literature, Physics, Chemistry, Biology, Geography, History, Psychology, Art and Design, Economics, and Computing. In Phase 2 (April 2014) of the present study, students were given the following question and example, “What were your A‐level subjects and results? Example answer: Biology – C, Chemistry – A, Psychology – A”. All students who participated in Phase 2 followed the example and reported their achieved GCE A‐Level results by listing the subjects and letter grade achieved for each subject. No students indicated that they retook any A‐levels. For each student, individual letter grades were converted to points and a sum of total points was calculated. The UCAS Tariff system was used to convert reported grades to points. At the time, the points were as follows: A* = 140 points, *A* = 120 points, *B* = 100 points, *C* = 80 points, *D* = 60 points, *E* = 40 points, and *F* = 0 points.

### 2.5 Possible confounding variables

All potential confounding variables were selected a priori. Depressive symptomology was controlled for based on the association between depression and cardiovascular stress reactivity (e.g., Brindle et al., 2013; Schiweck et al., 2019) and the association between depression and cognitive function (e.g., McDermott & Ebmeier, 2009; Scult et al., 2017). Depression was measured using the Hospital Anxiety and Depression Scale (HADS; Zigmond & Snaith, 1983). The HADS is comprised of 14 items, 7 measuring depression and 7 measuring anxiety. The HADS has good concurrent validity and test–retest reliability (Bramley et al., 1988; Herrmann, 1997), Cronbach’s alpha in the current sample = 0.73. Higher levels of cardiorespiratory fitness have been associated with higher levels of academic achievement (e.g., Sardinha et al., 2016). Therefore, cardiorespiratory fitness was selected as a potential confounding variable. Cardiorespiratory fitness was assessed using a validated formula designed to assess fitness without exercise testing (Jurca et al., 2005; Mailey et al., 2010). The formula has previously been used in adolescent and young adult populations (Ginty, Phillips, Higgs, et al., 2012; Heaney et al., 2011; Williams et al., 2016). For the formula, women were assigned a 0 and men were assigned a 1. Average baseline HR was used for resting HR. Participants were asked to categorize their physical activity levels as 1, 2, 3, 4, or 5 (1 = significant inactivity and 5 = participation in brisk exercise for over 3 h per week) and then were assigned scores of 0.00, 0.32, 1.06, 1.76, and 3.03, respectively (Jurca et al., 2005). Cardio‐respiratory fitness in METS was estimated using the following formula, ([gender^1^] × 2.77) − ([age] × 0.10) − ([BMI] × 0.17) − ([resting HR] × 0.03) + (physical activity score) + 18.07 (Jurca et al., 2005). Socioeconomic status (SES) has been associated with cognitive function and cardiovascular stress reactivity (e.g., Boylan et al., 2018; Lawson et al., 2018; Sirin, 2005). Parental SES was characterized by the occupation of the head of the parental household using the registrar general’s occupation classification system. It ranges from 1 to 7 (in the current study, 1 = “professional” and 7 = “unskilled worker”).

### 2.6 Laboratory procedure

Participants provided informed consent and completed questionnaires in a quiet room. After questionnaires were completed, participants were asked to lay in a semi lateral decubitus position on a standard bed with pillows for support and comfort. ECG electrodes were applied and a blood pressure cuff was attached to the right arm. The Doppler echocardiograph probe was then positioned and the experimenter assessed the quality of the image. This was followed by a 10‐min adaptation period and a 10‐min formal resting baseline period during which the participants rested quietly. Participants were then read instructions for the PASAT and completed a brief practice to ensure they understood the task. Participants engaged in the 10‐min stress task followed by a 10‐min recovery period (recovery data not reported here). Cardiovascular measurements were averaged separately for each formal 10‐min phase (baseline, stress). Immediately after the PASAT, participants completed a brief questionnaire regarding task stressfulness, difficulty, and their level of engagement during the task. Participants responded on a standard Likert scale ranging from 0 to 6 (0 = “not at all” and 6 = “extremely”).

### 2.7 Statistical analyses

Differences in GCE A‐Level total scores between men and women and different ethnicities were examined using one‐way ANOVA. The relationship between GCE A‐Level total scores and age, baseline cardiovascular values, SES, HADS depression score, cardiorespiratory fitness, baseline cardiovascular levels, self‐reported task stressfulness, self‐reported task difficulty, and self‐reported task engagement were examined using Pearson’s bivariate correlations. Repeated‐measures ANOVAs, using baseline and task values, were undertaken to confirm that the PASAT perturbed cardiovascular activity. Partial eta squared (*η* ^2^) was used as a measure of effect size.

Cardiovascular stress reactivity was computed as: cardiovascular stress average—cardiovascular baseline average for each cardiovascular variable. A series of unadjusted linear regressions (separate regression for each cardiovascular variable) were undertaken to determine whether cardiovascular stress reactivity predicted A‐level performance. A series of separate hierarchical linear regressions were undertaken to determine if whether any effects that emerged from the primary analyses withstood possible adjustment for confounding variables. The possible confounders selected were age, ethnicity, sex, SES, HADS depression, cardiorespiratory fitness, baseline cardiovascular levels, self‐reported task stressfulness, self‐reported task difficulty, and self‐reported task engagement. Covariates were selected a priori given associations with cardiovascular reactivity and/or cognitive function (Harrell, 2000; Steyerberg et al., 2001). Lastly, a series of post‐hoc hierarchical linear regression analyses were conducted with the additional covariate of school attended.

## 3 RESULTS

### 3.1 Total A‐Level score

Mean (*SD*) total GCE A‐Level performance score was 340.30 (114.55); total scores ranged from 40 points to 700 points. Students from higher SES backgrounds and with lower levels of depression had higher GCE A‐Level total scores, *r* (133) = −0.208, *p* = .016 and *r* (133) = −0.257, *p* = .003, respectively. There were no statistically significant associations between age, gender, ethnicity, or cardiorespiratory fitness with total A‐Level performance. Similarly, there were no associations between self‐reported difficulty, stress, or engagement ratings of the acute psychological laboratory stress task with total A‐Level performance. There were no associations between resting cardiovascular activity on any of the variables with total A‐Level score. All variables remained in final analyses, as they were selected a priori because they are well‐established predictors of the outcome variables (Harrell, 2000; Steyerberg et al., 2001).

### 3.2 Cardiovascular stress reactivity and Self‐Reported task ratings

Two‐way (baseline, task) repeated measures ANOVA indicated that on average, the PASAT significantly increased cardiovascular activity for all cardiovascular variables. The mean baseline and reactivity values, and statistical outcomes are presented in Table 2. On a scale of 1 (lowest) to 6 (highest), participants reported being generally engaged (*M* = 4.10, *SD* = 1.27), found the task to be difficult (*M* = 4.23, *SD* = 1.04), and found the task to be stressful (*M* = 4.53, *SD* = 1.07).

**TABLE 2:** Mean (SD) cardiovascular activity at baseline and stress

	Baseline	Stress	F	*p*	*η* ^2^
Heart rate (bpm)	74.46 (12.29)	91.73 (14.55)	415.13	<.001	0.759
Systolic blood pressure (mmHg)	109.71 (9.74)	123.84 (12.78)	369.73	<.001	0.738
Diastolic blood pressure (mmHg)	67.10 (6.02)	75.88 (8.04)	319.96	<.001	0.710
Cardiac output (L/min)	5.29 (0.93)	7.03 (1.51)	260.83	<.001	0.705
Stroke volume (mL)	73.72 (10.74)	76.94 (11.47)	26.57	<.001	0.196

*Notes*: Repeated measures ANOVAs reveal that stress is significantly greater than baseline for all cardiovascular variables.

### 3.3 Total A‐Level score and cardiovascular stress reactivity

**FIGURE 1:**
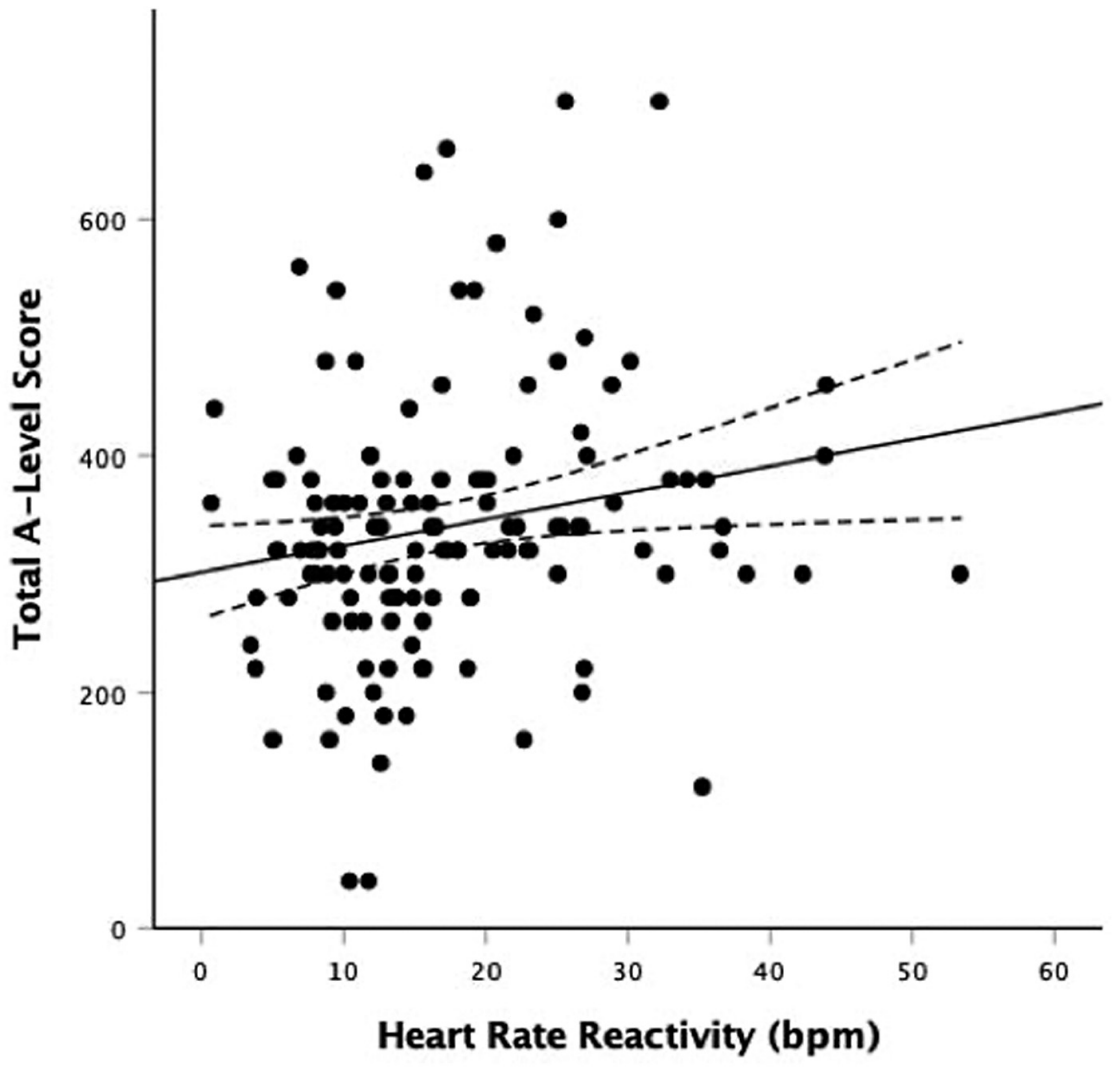
Scatterplot of the unadjusted relationship between heart rate reactivity (bpm) to acute psychological stress and A‐level scores. Solid line represents line of best fit (linear); dotted lines represent upper and lower 95% confidence intervals

**FIGURE 2:**
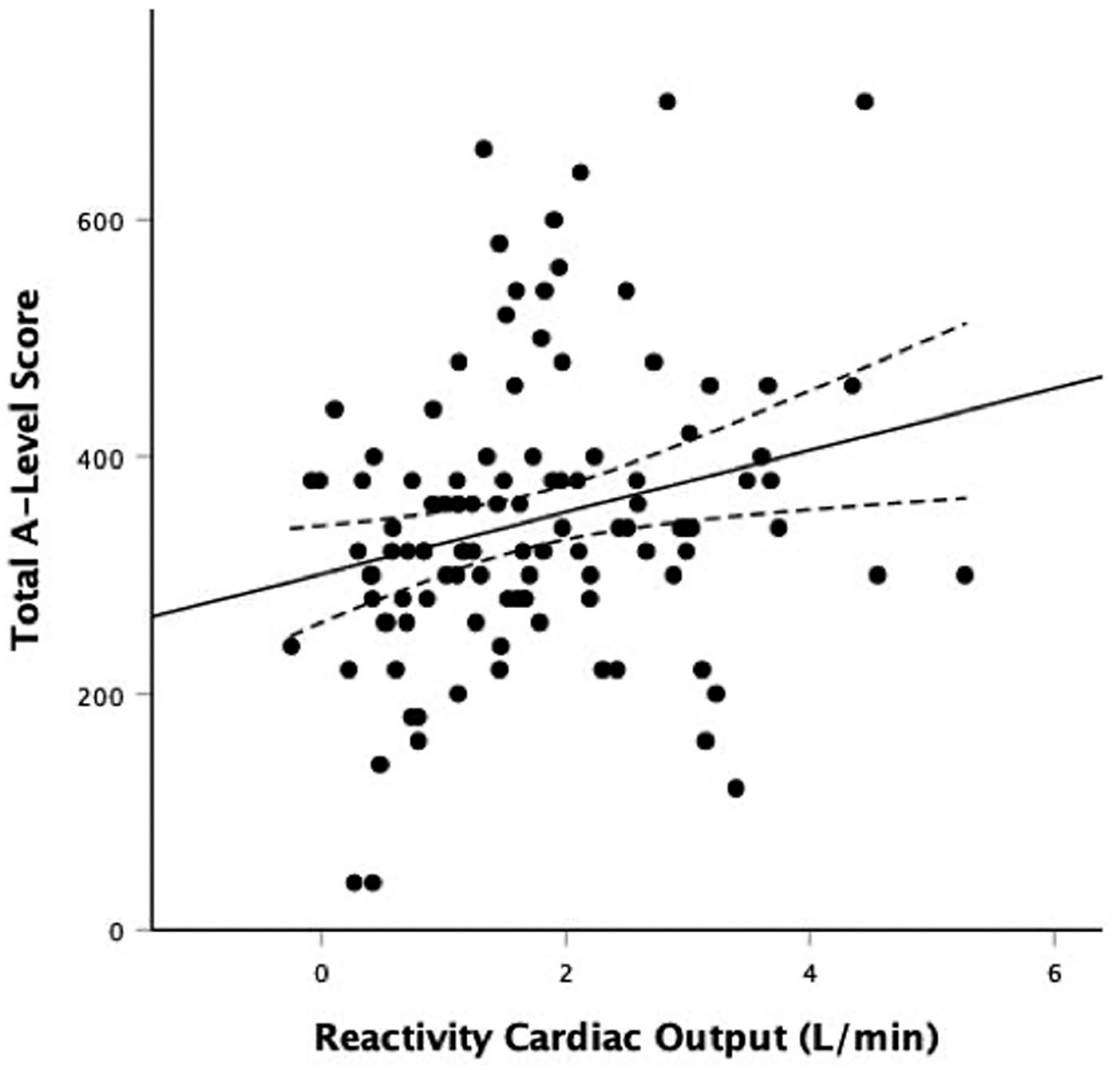
Scatterplot of the unadjusted relationship between cardiac output reactivity (L/min) to acute psychological stress and A‐level scores. Solid line represents line of best fit (linear); dotted lines represent upper and lower 95% confidence intervals

A series of regression analyses examined if cardiovascular reactivity (independent variable) was significantly associated with total A‐Level performance (dependent variable). In the first model, with no adjustment, HR reactivity was associated with total A‐Level performance, *β* = 0.192, *p* = .027, *R* ^2^ = 0.037; individuals with lower HR reactivity had lower A‐Level performance. Similarly, CO was associated with total A‐Level performance, *β* = 0.243, *p* = .011, Δ*R* ^2^ = 0.059; individuals with lower CO reactivity had lower A‐Level performance. There were no statistically significant associations of SV reactivity (*β* = 0.170, *p* = .075, *R* ^2^ = 0.029), SBP reactivity (*β* = 0.058, *p* = .506, *R* ^2^ = 0.003), or DBP reactivity (*β* = −0.016, *p* = .859, *R* ^2^ = 0.000) with A‐Level performance. In regression analyses that adjusted for baseline cardiovascular activity, age, gender, ethnicity, SES, HADS depression score, cardiorespiratory fitness, self‐reported task engagement, self‐reported task difficulty, and self‐reported task stressfulness in step 1, and respective cardiovascular reactivity indices in step 2, HR reactivity and CO reactivity continued to be associated with A‐Level scores, *β* = 0.189, *p* = .034, Δ*R* ^2^ = 0.032 and *β* = 0.277, *p* = .005, Δ*R* ^2^ = 0.067, respectively (see Tables 3 and 4 for fully adjusted regression models and Figures 1 and 2 for unadjusted scatter plots). HR reactivity explained an additional 3.2% of the variance for total A‐Level performance score and CO reactivity explained an additional 6.7% of the variance in final models. The associations were in the same directions with lower cardiovascular reactivity being associated with lower total A‐Level performance. Results remained statistically significant when conducting post‐hoc sensitivity analyses additionally adjusting for school attended. SV(*β* = 0.177, *p* = .068, *R* ^2^ = 0.029), SBP (*β* = 0.036, *p* = .685, *R* ^2^ = 0.001), and DBP (*β* = 0.015, *p* = .863, *R* ^2^ = 0.000) were not statistically significantly associated with A‐Level performance score in the fully adjusted models.

**TABLE 3:** Predictors of Total GCE A‐level score in the fully adjusted heart rate reactivity model

	*β*	*p*	Δ*R* ^2^	Lower CI	Upper CI
*Step 1*					
Age	0.126	.154		−13.104	82.005
Gender	−0.162	.149		−121.243	18.686
Ethnicity	0.089	.305		−6.822	21.599
SES	−0.066	.475		−16.894	7.909
HADS‐depression	−0.167	.074		−12.192	0.578
Task engagement	0.055	.534		−10.855	20.85
Task difficulty	0.228	.018		4.399	45.491
Task stressfulness	−0.062	.517		−26.753	13.520
Cardiorespiratory fitness	0.182	.138		−3.567	25.408
Baseline heart rate	0.003	.973	0.154	−1.810	1.872
*Step 2*					
Heart rate reactivity	0.189	.034	0.032	0.172	4.229

**TABLE 4:** Predictors of Total GCE A‐level score in the fully adjusted cardiac output reactivity model

	*β*	*p*	Δ*R* ^2^	Lower CI	Upper CI
*Step 1*					
Age	0.128	.187		−18.217	91.950
Gender	−0.134	.244		−129.382	33.295
Ethnicity	0.074	.440		−9.843	22.478
SES	−0.061	.555		−18.978	10.258
HADS‐depression	−0.156	.132		−12.805	1.708
Task engagement	0.049	.609		−13.346	22.647
Task difficulty	0.258	.014		6.195	52.473
Task stressfulness	−0.064	.526		−30.010	15.443
Cardiorespiratory fitness	0.177	.138		−3.691	26.278
Baseline cardiac output	−0.087	.379	0.162	−36.421	13.985
*Step 2*					
Cardiac output reactivity (L/min)	0.277	.005	0.067	9.349	50.072

## 4 DISCUSSION

The present study examined the association between cardiovascular stress reactivity measured in the laboratory and academic performance, as indexed by total scores on A‐levels. A‐Levels are an indirect measure of cognitive ability (McManus et al., 2005) and a marker for multidimensional forms of cognitive function such as critical thinking skills (O’Hare & McGuinness, 2009) and general knowledge (O’Hare & McGuinness, 2009). In line with our hypothesis, higher levels of HR and CO stress reactivity were associated with better A‐Level performance. These associations remained statistically significant after adjusting for age, gender, ethnicity, SES, depressive symptomology, cardiorespiratory fitness, respective baseline cardiovascular activity, self‐report task engagement, self‐report task difficulty, and self‐report task stressfulness.

The positive associations between HR reactivity and A‐level performance are in line with previous research in relatively large samples (*N*’s > 340) demonstrating higher HR reactivity is associated with better cognitive performance across a range of populations and cognitive assessments (Gao et al., 2015;Ginty, Phillips, Der, Deary, & Caroll, 2011; Ginty, Phillips, Der, Deary, & Carroll, 2011; Ginty, Phillips, Roseboom, et al., 2012; Lin et al., 2014), but at odds with one study in a small sample (*N* = 32) demonstrating no association between HR reactivity and cognitive function (Hendrawan et al., 2012). The effect sizes for the association of HR reactivity with future A‐Level performance are in the same range as effect sizes reported between HR reactivity and cognitive function in youth (Gao et al., 2015), but slightly higher than the effect sizes for HR reactivity and cognition in mid‐life and older adult samples (Ginty, Phillips, Der, Deary, & Caroll, 2011; Ginty, Phillips, Der, Deary, & Carroll, 2011; Ginty, Phillips, Roseboom, et al., 2012; Lin et al., 2014). While research supports a bi‐directional relationship between HR reactivity and cognitive function (Ginty, Phillips, Der, Deary, & Caroll, 2011; Ginty, Phillips, Der, Deary, & Carroll, 2011), longitudinal studies are needed to disentangle the strength of the relationship across the lifespan. It is possible that cognitive function and HR reactivity are more strongly related in early life. However, A‐Level performance may be measuring more than just cognitive ability or cognitive function and this may possibly be contributing to the larger effect sizes.

To perform well on A‐Levels, students need to have high levels of cognitive function and also need to demonstrate high levels of motivation and a commitment to learning (McManus et al., 2005). A now established body of research suggests that higher cardiac stress reactivity may be associated with higher levels of motivation and behavioral engagement (Chauntry et al., 2019; Ginty et al., 2015; Ginty et al., 2020; Messay & Marsland, 2015; Whittaker & Chauntry, 2021). In other words, low or blunted levels of cardiac stress reactivity may be associated with lower levels of behavioral engagement. Indeed, blunted cardiovascular stress reactivity has been associated with a range of clinical and non‐clinical negative behavioral outcomes that involve forms of dysfunction in motivation, such as depression (Brindle et al., 2013; Carroll et al., 2007; de Rooij et al., 2010; Phillips et al., 2011; Salomon et al., 2009; Salomon et al., 2013; Schiweck et al., 2019; Schwerdtfeger & Rosenkaimer, 2011) and addictive behaviors (alʼAbsi, 2018; alʼAbsi et al., 2021; Heaney et al., 2011; Lovallo, 2007; Milivojevic & Sinha, 2018). Similarly, neuroimaging studies have shown blunted cardiovascular responses to stress are associated with less activation in brain regions associated with goal‐directed behavior and cognitive function, such as the medial prefrontal cortex, anterior cingulate, and amygdala (Bush et al., 2000; Bush et al., 2002, 2008; Critchley et al., 2003; Gianaros et al., 2005; Ginty et al., 2013; Ginty, 2013; Paus, 2001; Wager et al., 2009). Therefore, it is plausible that the effect sizes between reactivity and a “real world” task that encompasses aspects of cognitive function, but also requires high levels of behavioral engagement and motivation to study, would be higher than the relationship between reactivity and a simple laboratory cognitive challenge.

While, to our knowledge, no study has explicitly examined laboratory‐based cardiovascular stress reactivity with performance on a “real world” cognitive task, one previous study examined the association between reactivity and performance on a mock A‐Level examination. In a fully applied study, participants’ HR was measured during normal class time and then again during a mock A‐Level French exam. HR reactivity, for each participant, was defined as a ratio score of: test HR activity/normal class HR activity. Results demonstrated a trend between higher HR ratio and performance on the test; individuals with higher HR activity during the examination compared to during normal class time performed better on the mock examination (Daly et al., 2011). While the relationship was not statistically significant, the effect size was large and non‐significance may be attributed to the small sample size (*N* = 39). Although the Daly et al., 2011 study was conducted in a completely applied setting, defined HR reactivity slightly differently, and used a mock examination, the results are generally supportive of the present findings.

Research examining the association between BP reactivity and cognitive function has produced mixed results. Some have reported a negative association between BP and cognitive function (Brown et al., 2009; Wawrzyniak et al., 2016), others reporting a positive association (Ginty, Phillips, Roseboom, et al., 2012; Yano et al., 2016), and others reporting no association in fully adjusted models (Ginty, Phillips, Der, Deary, & Caroll, 2011; Ginty, Phillips, Der, Deary, & Carroll, 2011). In the present study, there were no statistically significant associations between SBP or DBP with A‐Level performance in unadjusted or adjusted models. This is the first study to utilize more comprehensive hemodynamic measurements of cardiovascular reactivity (i.e., measures of SV and CO). There was no statistically significant relationship between SV and cognitive function; however, there was a trend suggesting that higher levels of SV reactivity change were associated with higher A‐level performance. In fully adjusted models, SV reactivity, although not statistically significant, accounted for 2.9% of the variance in A‐level scores. Indeed, SV reactivity explained a larger percent of the variance than HR reactivity did in studies examining the association between HR reactivity and cognitive function in middle age and older adults (e.g., Ginty, Phillips, Der, Deary, & Carroll, 2011; Lin et al., 2014). CO reactivity explained 6.7% of the variance of A‐Level scores in fully adjusted models. The high level of variance explained by CO reactivity is unsurprising given CO is a product of both SV and HR. Examination of the cardiovascular reactivity variables that predicted A‐Levels would suggest that the relationship between reactivity and cognitive function may be primarily cardiac in nature, as opposed to vascular. Given that HR, CO, and SV are modulated primarily by changes in the sympathetic (e.g., β‐adrenergic) and parasympathetic nervous systems (Gordan et al., 2015), it may be that stress‐related changes in nervous system activity, in some way, are associated with the relationship between stress reactivity and cognition. However, this remains to be empirically tested, as the current study was unable to probe underlying mechanisms of cardiac change. Future research should aim to include additional measurements that capture both sympathetic nervous system and parasympathetic nervous system activity (Brindle et al., 2014).

The present study is not without limitations. First, while the study is prospective in design, it is still observational and causality cannot be determined (Christenfeld et al., 2004). It is possible results are being influenced by unknown variables. For example, SES is associated with secondary school academic performance in the UK (Morris et al., 2016; Strand, 2015). Interestingly, a recent meta‐analysis reported that lower levels of SES were associated with lower reactivity to cognitive stressors (Boylan et al., 2018). Given the relationship between SES and reactivity and SES and academic performance, individual differences in SES may have contributed to the effect sizes in the study. However, the statistically significant associations between HR reactivity and CO reactivity and A‐level performance remained even when adjusting for SES. Similarly, results withstood adjustment for other possible important confounding variables, such as depressive symptomology, task engagement, perceptions of the task, cardiorespiratory fitness, baseline values, age, and gender. Second, A‐level subjects studied (e.g., Biology, Psychology, Art) may have influenced grades obtained. Each participant took at least 3 A‐level subjects, resulting in 110 different combinations of subjects. However, having results of all A‐levels, rather than just one specific subject, strengthens the generalizability of the present study. Third, it could be argued that completing a laboratory task requires engagement and motivation and those with lower levels of cardiovascular reactivity were simply not engaged in the laboratory acute psychological stress task. However, all analyses survived adjustment for reported difficulty, stressfulness, and engagement during the acute psychological stress task. The associations between low reactivity and motivation appear to be related to more of a prolonged or chronic disengagement and motivation, rather than acute (Ginty et al., 2020). In support of this, there were also no associations between reported task difficulty, stressfulness, and engagement with total A‐level scores. This level of chronic disengagement may possibly be impacting A‐level performance through differences in motivation to learn, studying, and the seriousness through which one takes their academics. However, the present study did not include an objective measure of task engagement. In order to fully disentangle the relationship between cardiovascular reactivity and motivational disengagement, future studies need to include methods to comprehensively and objectively measure task engagement that are independent of performance. Fourth, students were recruited from 28 different schools in the Birmingham area to ensure a diverse and representative sample. It may be possible that participants are nested within‐schools and not truly independent. However, A‐level examinations are standardized across the country so school would not have impacted the examination content nor rigor of examination scoring and post‐hoc analyses controlling for school did not change results. Fifth, students were asked to self‐report their A‐level results approximately 6.5 months after receiving them, meaning there is always the potential for some inaccuracies or self‐report bias. However, all students responded to the prompt and listed specific subjects and marks for each subject. Sixth, other factors, rather than pure cognitive ability, contribute to A‐level performance. For example, the correlation between general intelligence and academic performance in adolescents is, on average, about 0.50 (Deary et al., 2007; Neisser et al., 1996; Morris et al., 2016; Rhode & Thompson, 2007;). This suggests A‐level performance may be influenced by both intrapersonal and situational factors. Therefore, the present study should be considered an extension of the work on cardiovascular reactivity and cognitive ability, rather than a replication.

In conclusion, higher cardiac stress reactivity measured in the laboratory was associated with better A‐level performance. Our results support most, but not all, previous research examining associations between cardiovascular stress reactivity and different domains of cognitive function. The present study extends previous work by including more comprehensive cardiovascular measurements and utilizing a “real world” outcome. Results also support an established body of literature demonstrating that high cardiovascular responses to acute psychological stress are not always detrimental and that low or blunted responses to acute psychological stress are not necessarily adaptive and may be associated with a host of adverse behavioral and health outcomes (for reviews see: Carroll et al., 2017; Turner et al., 2020).

## AUTHOR CONTRIBUTIONS

**Annie Ginty:** Conceptualization; data curation; formal analysis; funding acquisition; investigation; methodology; project administration; visualization; writing – original draft; writing – review and editing. **Alexandra T Tyra:** Writing – review and editing. **Danielle A. Young:** Writing – review and editing. **Ryan C Brindle:** Methodology; project administration; visualization; writing – review and editing. **Susanne R de Rooij:** Conceptualization; writing – review and editing. **Sarah E Williams:** Conceptualization; investigation; methodology; visualization; writing – review and editing.

## Footnotes

^1^
0 = women, 1 = men
